# Enhanced microbial production of pyridoxine (Vitamin B_6_) in *Bacillus subtilis* via pathway and process optimization

**DOI:** 10.1016/j.synbio.2025.09.014

**Published:** 2025-09-16

**Authors:** Ai-Tong Jiang, Guang-Qing Du, Xu-Yang Huang, Zheng-Zi Ji, Si-Riguleng Qian, Lin-Xia Liu, Da-Wei Zhang

**Affiliations:** a School of Biological Engineering, Dalian Polytechnic University, Dalian, China; b Tianjin Institute of Industrial Biotechnology, Chinese Academy of Sciences, Tianjin, China; c National Center of Technology Innovation for Synthetic Biology, Tianjin, China; d State Key Laboratory of Engineering Biology for Low-Carbon Manufacturing, Tianjin Institute of Industrial Biotechnology, Chinese Academy of Sciences, Tianjin, China; e University of Chinese Academy of Sciences, Beijing, China

**Keywords:** Pyridoxine, *Bacillus subtilis*, DXP-Independent pathway, Medium optimization

## Abstract

Vitamin B_6_ refers to a family of water-soluble B vitamin, which is essential for various physiological functions, including amino acid metabolism, neurotransmitter synthesis, and hemoglobin synthesis. The biosynthesis of pyridoxine (PN), a commercial form of vitamin B_6_, through microbial fermentation has garnered widespread attention owing to its environmentally friendly and safe production methods, as well as its mild reaction conditions. However, the low yield of natural strains limits their application. This study focused on constructing a high-yielding strain of PN through pathway engineering and process optimization. Firstly, five key deoxyxylulose-5-phosphate-dependent pathway genes (*epd*, *pdxB*, *serC*, *pdxA*, and *pdxJ*) were overexpressed in *Bacillus subtilis* ARTP, which improved the PN titer by 3.2-fold to 2.9 mg/L. Subsequently, *pdxST* genes involved in the DXP-independent pathway were screened from various strains. Ribosome binding site (RBS) sequences were optimized to regulate their expression, which further increased the PN titer to 24.6 mg/L. Finally, systematic medium optimization was identified as a critical strategy for enhancing PN biosynthesis, leading to a remarkable 1.8-fold improvement in PN production. Under optimized fermentation conditions, the engineered strain achieved a PN titer of 174.6 mg/L in fed-batch fermentation, which represents the highest level reported to date in *B*. *subtilis*. Overall, this study presents an effective strategy combining pathway engineering and medium optimization for significantly improving PN production, offering valuable insights for the industrial development of PN biosynthesis.

## 1 Introduction

Vitamin B_6_, a water-soluble vitamin comprising pyridoxine (PN), pyridoxal (PL), pyridoxamine (PM), pyridoxine 5′-phosphate (PNP), pyridoxal 5′-phosphate (PLP), and pyridoxamine 5′-phosphate (PMP), functions as a coenzyme in a variety of enzymatic reactions related to amino acid, glucose, and lipid metabolisms [[1], [2], [3]]. Vitamin B_6_ is widely used in the pharmaceutical industry and as a nutritional additive in food and feed due to its roles in enhancing immune function and neurological health [[4], [5], [6], [7], [8]]. Additionally, Vitamin B_6_ contributes to the alleviation of inflammation and oxidative stress, making it highly valuable in healthcare and medical applications [4,5,7,9]. Currently, the industrial production of vitamin B_6_ mainly relies on chemical synthesis, which often involves harsh reaction conditions, toxic reagents, and environmental concerns [[10], [11], [12], [13]]. To address these issues, the development of safe and eco-friendly microbial biosynthesis routes has attracted increasing attention [14]. Although the biosynthetic pathway of PN, the commercial form of vitamin B_6_, has been elucidated in several microorganisms, the natural yield remains insufficient to meet industrial demands [11,15]. Therefore, it is imperative to further improve the biosynthetic efficiency through metabolic engineering and synthetic biology strategies to enable sustainable and scalable production of PN.

**Fig. 1:**
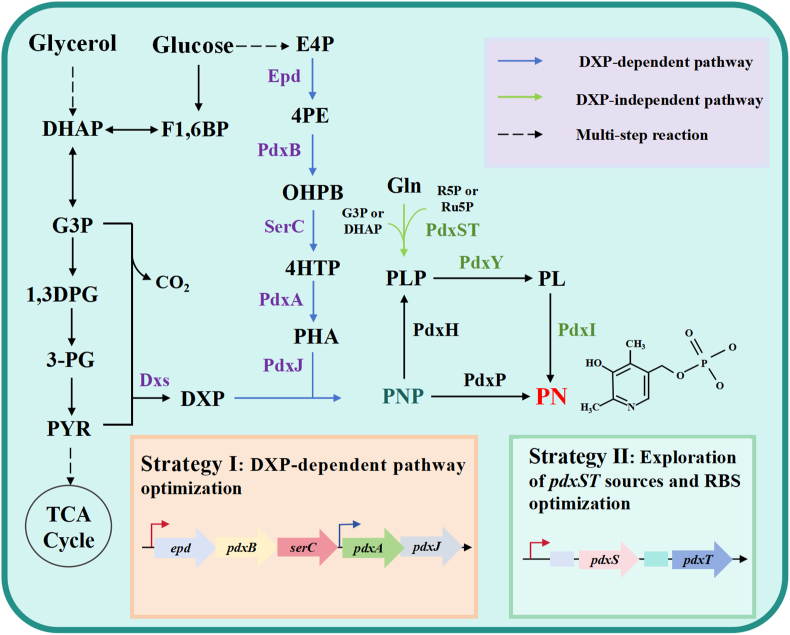
**The biosynthetic pathway of pyridoxine (PN)**. The blue arrow indicates the DXP-dependent PN biosynthetic route in *B*. *subtilis*. Green arrows represent heterologous pathways independent of DXP. The dotted line represents a multi-step response. Enzymes: Epd: erythrose 4-phosphate dehydrogenase, PdxB: 4-phosphoerythronate dehydrogenase, SerC: 3-phosphoserine aminotransferase, PdxA: 4-phosphohydroxy-l-threonine dehydrogenase, PdxJ: PNP synthase, *dxs*: 1-deoxyxylulose 5-phosphate synthase, PdxP: PNP phosphatase, PdxH: PNP oxidase, PdxY: PL kinase, PdxI: PL reductase. Metabolites: E4P: erythrose 4-phosphate, 4PE: 4-phosphoerythronate, OHPB: 2-oxo-3- hydroxy-4-phosphobutanoate, 4HTP: 4-phosphohydroxy-l-threonine, PHA: 3-phosphohydroxy-1-aminoacetone, DHAP: dihydroxyacetone phosphate, G3P: glyceraldehyde 3-phosphate, 1,3DPG: 1,3-bisphospho-d-glycerate, PYR: pyruvate, AcCoA: acetyl-CoA, Gln: glutamine, R5P: ribose 5-phosphate, Ru5P: d-ribulose 5-phosphate.

The biosynthesis of PN in microorganisms involves two distinct pathways: the deoxyxylulose-5-phosphate (DXP)-dependent pathway and the DXP-independent pathway [2]. In *Escherichia coli* and many γ-proteobacteria, the DXP-dependent pathway is the native route. It begins with the condensation of glyceraldehyde-3-phosphate (G3P) and pyruvate catalyzed by 1-deoxy-d-xylulose-5-phosphate synthase (Dxs) to produce DXP. In parallel, erythrose-4-phosphate (E4P) is converted into 4-phosphohydroxy-l-threonine (4HTP) via erythrose-4-phosphate dehydrogenase (Epd), phosphoserine aminotransferase (SerC), and pyridoxine 5′-phosphate synthase B (PdxB). These two branches converge through the action of PNP synthase (PdxJ) and PNP oxidase (PdxH), yielding PLP. In *E. coli*, this pathway is tightly coordinated with the pentose phosphate pathway and glycolysis, but flux can be limited by enzyme catalytic efficiency and competition for precursors such as E4P and G3P (Fig. 1) [2]. In contrast, *Bacillus subtilis* and many Gram-positive bacteria lack the native DXP-dependent pathway and instead employ the DXP-independent pathway, which bypasses DXP entirely and is catalyzed by the PdxS-PdxT hetero-oligomeric complex. In this system, PdxT acts as a glutaminase, generating ammonia from glutamine, while PdxS catalyzes the condensation of ribose-5-phosphate (R5P) or ribulose-5-phosphate with dihydroxyacetone phosphate (DHAP) in the presence of ammonia to directly produce PLP. This pathway is naturally integrated into *B. subtilis* central metabolism and avoids the accumulation of toxic intermediates such as 4HTP, but its catalytic turnover can be limited by subunit imbalance, feedback inhibition, or cofactor availability. To enhance the metabolic flux of the DXP-dependent pathway, multiple strategies have been employed, including the overexpression of key enzymes PdxA and PdxJ, optimization of the ribosome binding site (RBS) of corresponding genes, and rational engineering of the enzyme Epd. Moreover, increasing the intracellular availability of precursors like E4P and glyceraldehyde-3-phosphate (G3P) is also critical for improving PN biosynthesis by boosting overall pathway throughput. In contrast, the DXP-independent pathway relies on the PdxST enzyme complex, which directly synthesizes pyridoxal 5′-phosphate (PLP), the biologically active form of vitamin B_6_. Within this system, PdxT functions as a glutaminase, converting glutamine into ammonia, which is subsequently utilized by PdxS. PdxS catalyzes the condensation of ammonia with ribose 5-phosphate (or ribulose 5-phosphate) and either G3P or dihydroxyacetone phosphate (DHAP) to produce PLP (Fig. 1) [[16], [17], [18]]. Although the DXP-independent pathway involves fewer enzymes, it is limited by several factors, including the requirement for precise enzyme complex assembly, dependence on intra-complex ammonia transfer, and inherently low catalytic efficiency [4,16]. These constraints significantly hinder its application for high-level production of PLP or the biosynthesis of other vitamin B_6_ derivatives in microbial hosts (Fig. 1) [19]. In living organisms, the vitamin B_6_ salvage pathway is universally present, regardless of whether they possess a de novo biosynthetic route. Organisms lacking the de novo pathway can take up PN, PL, PM from the environment and interconvert them into the six natural vitamers of vitamin B_6_. The salvage pathway is mainly mediated by two enzymes: pyridoxal kinase PdxK and pyridoxamine phosphate oxidase PdxH. PdxK phosphorylates PN, PL, and PM to generate PNP, PLP, and PMP, respectively. Both PNP and PMP can subsequently be oxidized by PdxH to form PLP, the biologically active form of vitamin B_6_. In addition, dephosphorylation of PNP, PLP, and PMP is catalyzed by specific phosphatases, such as PLP phosphatase, which belongs to the haloacid dehalogenase (HAD) superfamily. The salvage pathway provides an essential mechanism for maintaining vitamin B_6_ homeostasis by enabling uptake, phosphorylation, interconversion, and recycling of B_6_ vitamers [20].

To date, several efforts have been made to enhance microbial production of PN through metabolic engineering and systems biology strategies. Metabolic engineering of *E*. *coli* has demonstrated the potential of microbial fermentation for high-level PN biosynthesis. By enhancing the deoxyxylulose 5-phosphate (DXP)-dependent vitamin B_6_ pathway through targeted overexpression and rational protein engineering of key enzymes such as PdxA, PdxJ, Dxs, and Epd, along with iterative optimization of ribosome binding sites (RBSs), a PN titer of up to 1.4 g/L with a productivity of 29.16 mg/L/h was achieved in fed-batch fermentation. These studies underscore the remarkable efficiency of the engineered DXP-dependent pathway in *E. coli*, which enabled gram-level PN production through precise enzyme optimization and regulatory fine-tuning [21]. However, despite its metabolic robustness, *E. coli* poses limitations for industrial applications due to strict regulatory constraints in food-related and pharmaceutical production. As an alternative, *B*. *subtilis*, a generally recognized as safe (GRAS) microorganism, offers significant advantages as a microbial chassis. Previous studies have focused on heterologously introducing the DXP-dependent pathway into *B. subtilis* [[22], [23], [24], [25], [26], [27]]. Although supplementation with intermediates such as 4HTP and deoxyxylulose (DX) led to improved PN titers (up to 65 mg/L), the overall productivity remained substantially lower than that achieved in *E. coli*. Alternatively, the DXP-independent pathway has also been explored in *B. subtilis* by overexpressing the *pdxST* gene cluster, which enables direct biosynthesis of PLP. This approach showed promise by bypassing some of the limitations of the DXP-dependent route, although enzyme complex assembly and low catalytic efficiency still restrict its effectiveness [15]. Given the contrasting features of the two biosynthetic pathways, it is important to explore both the DXP-dependent and DXP-independent strategies in *B. subtilis* to assess which approach is more effective for PN biosynthesis. In addition, the compatibility between the engineered metabolic pathways and the fermentation medium may also significantly impact PN production and should be carefully evaluated.

In this study, we engineered *B*. *subtilis* to express both optimized DXP-dependent and DXP-independent pathways for PN biosynthesis. The heterologous DXP-dependent pathway was reconstructed by overexpressing key genes, including *epd*, *pdxB*, *serC*, *pdxA*, and *pdxJ*, leading to a 3.2-fold increase in PN titer compared to the wild-type strain. Building on this, three PdxST isoenzymes from different microbial sources were further introduced to evaluate the performance of the DXP-independent pathway within the same host. Comparative analysis demonstrated that the DXP-independent route showed better compatibility with the *B. subtilis* chassis and resulted in significantly enhanced PN production. To further improve yield, medium optimization was carried out through a combination of single-factor experiments and orthogonal design, resulting in a refined formulation that significantly enhanced PN production, with the titer reaching 45.02 mg/L. Under fed-batch fermentation in a 5-L bioreactor, the engineered strain achieved a final PN titer of 174.6 mg/L, representing the highest level reported in *B. subtilis* to date. These results highlight the potential of *B. subtilis* as a robust and sustainable microbial chassis for the production of PN via rational pathway selection and process optimization.

## 2 Materials and methods

### 2.1 Bacterial strains, plasmids, media, and growth conditions

The bacterial strains and plasmids used in this study, including both wild-type and genetically engineered variants, are listed in Table S1. *E. coli* DH5α was employed as the cloning host for plasmid construction, and *B*. *subtilis* ATRP served as the chassis strain for PN production [28]. *E. coli* strains were cultured in Luria-Bertani (LB) broth or on LB agar plates containing 2 % (w/v) agar for solid media. When necessary, kanamycin was added at a final concentration of 50 μg/mL for plasmid maintenance. All strains were cultivated at 37 °C with shaking at 200 rpm. Glycerol stocks (20 %, v/v) were stored at −80 °C and revived by streaking onto LB agar plates. Four types of media, J1, FM1.4, SRG, and SVY, were used for fermentation to evaluate their effect on PN production. The J1 medium used for fermentation contained the following components (per liter): 12 g glycerol, 4 g glucose, 2 g succinic acid, 8 g yeast extract, 7 g acid-hydrolyzed casein, 5 g NaCl, 0.2 g MgSO_4_·7H_2_O, 0.01 g MnSO_4_, 0.01 g FeSO_4_, and 100 mM Na_2_HPO_4_·12H_2_O. The FM1.4 medium consisted of (per liter): 15 g glycerol, 1 g glucose, 10 g Bacto Peptone, 5 g yeast extract, 0.2 g MgSO_4_·7H_2_O, 0.01 g FeSO_4_·7H_2_O, 0.01 g MnSO_4_·5H_2_O, and 100 mM Na_2_HPO_4_, pH 6.5 [29]. The SRG medium was prepared by mixing the following: 100 mL of a 10 × Spizizen minimal salts solution (containing 20 g (NH_4_)_2_SO_4_, 140 g K_2_HPO_4_, 60 g KH_2_PO_4_, 10 g sodium citrate·1H_2_O, and 2 g MgSO_4_·7H_2_O), 10 mL of a 100 × SRG trace element solution (containing 12.5 g MgCl_2_·6H_2_O, 0.55 g CaCl_2_, 1.35 g FeCl_2_·6H_2_O, 0.1 g MnCl_2_·4H_2_O, 0.17 g ZnCl_2_, 0.043 g CuCl_2_·H_2_O, 0.06 g CoCl_2_·6H_2_O, and 0.06 g Na_2_MoO_4_·2H_2_O), 2 mL of 50 % (w/v) glucose, 36 mL of 25 % (w/v) raffinose, 10 mL of 10 % (w/v) yeast extract, and 842 mL of sterile distilled water [30]. The SVY medium contained (per liter): 20 g Difco Veal Infusion Broth, 5 g Difco Yeast Extract, 2 g (NH_4_)_2_SO_4_, 5 g sodium glutamate, and 30 g glucose [11]. For all media, the pH was adjusted to 6.5 before sterilization.

### 2.2 DNA manipulation and strain construction

The primers used for genome editing and gene overexpression are listed in Table S2. Chromosomal gene fragments were amplified by polymerase chain reaction (PCR) using either PrimeSTAR HS DNA Polymerase (Takara), depending on the specific requirements of the amplification. DNA fragments were assembled by overlap extension PCR to generate recombinant plasmids. The resulting constructs were initially screened via colony PCR and subsequently verified by Sanger sequencing to ensure sequence accuracy.

Plasmids were introduced into *B*. *subtilis* ATRP using the Spizizen transformation method. Briefly, *B. subtilis* cells were cultivated in GM1 minimal medium until the optical density at 600 nm (OD_600_) reached up to 0.8–1.0. Then, 0.5 mL of the culture was transferred into 5 mL of fresh GM1 medium and incubated for an additional 4 h at 37 °C with shaking (200 rpm). Subsequently, 0.7 mL of the culture was subcultured into 5 mL of GM2 medium and grown for 2 h under the same conditions to induce competence. Approximately 500 ng of plasmid DNA was mixed with 100 μL of competent cells, followed by static incubation at 37 °C for 90 min. Transformants were selected by plating on LB agar supplemented with the appropriate antibiotic. Positive transformants were identified by colony PCR and confirmed by DNA sequencing [31]. The sequences of the RBS driving protein translation with different predicted rates were designed using the online RBS calculator tool (https://salislab.net/software/). The optimized RBS sequences are listed in Table S3.

### 2.3 Fermentation

For shake-flask fermentation, A single colony was obtained by streaking from glycerol stocks onto LB agar plates and incubating overnight at 37 °C. This colony was used to inoculate 5 mL of LB medium in a test tube and cultured at 37 °C with shaking at 200 rpm for 12 h. The resulting seed culture was transferred into 250 mL baffled Erlenmeyer flasks containing 30 mL of fermentation medium with a final OD_600_ of 0.1 and incubated at 37 °C and 180 rpm. To further enhance PN production, key medium components (glycerol, acid hydrolyzed casein, MgSO_4_·7H_2_O, and MnSO_4_) affecting PN titer were optimized using an orthogonal experimental design to determine their optimal concentrations. Each factor was tested at three levels, and a L9(3^4^) orthogonal table was used for the experimental setup. Orthogonal tables are generated by the Orthogonal Experiment Assistant Ⅱ. The experiments were conducted in shake flasks with a cultivation temperature of 37 °C, a fermentation time of 120 h, and a shaking speed of 200 rpm. Samples were taken at 96 h for PN yield measurement. Each experimental condition was repeated three times, and PN production was quantified. The data were statistically analyzed using the Orthogonal Experiment Assistant to evaluate the effects of individual factors and their interactions on PN yield, and to identify the optimal level for each factor. The orthogonal table is shown in Table S4. The resulting optimized medium, designated J2, contained the following components (per liter): 15 g glycerol, 4 g glucose, 5 g acid-hydrolyzed casein, 5 g NaCl, 0.5 g MgSO_4_·7H_2_O, 0.05 g MnSO_4_, 0.01 g FeSO_4_, and 100 mM Na_2_HPO_4_·12H_2_O. The pH was also adjusted to 6.5 prior to sterilization.

For fed-batch fermentation, all experiments were carried out in a 5-L bioreactor. The strain was streaked on LB agar plates and incubated at 37 °C overnight. A single colony was inoculated into 5 mL of LB medium supplemented with 5 μL kan50 and cultured for 12 h. The resulting culture was then transferred into 1-L baffled Erlenmeyer flasks containing 200 mL of LB medium with 50 μg/mL kanamycin and incubated at 37 °C with shaking (220 rpm) until reaching mid-logarithmic phase, which was used as the seed culture, and the pH was controlled at 7.0 via automatic addition of phosphoric acid (H_3_PO_4_) and ammonia solution (NH_3_·H_2_O). Dissolved oxygen (DO) levels were maintained above 30 % saturation throughout the fermentation process by modulating the agitation speed and aeration rate. Initially, the aeration rate was set at 2.0 L/min, and the agitation speed was started at 200 rpm, gradually increasing to a maximum of 1,000 rpm as fermentation progressed. DO levels were monitored in real time, and a DO spike indicated substrate depletion. At this point, DO-coupled feeding was initiated to maintain metabolic activity and improve product accumulation.

### 2.4 Statistical analysis and significance annotation

Statistical analyses were conducted using SPSS software. Comparisons among multiple groups were performed using one-way analysis of variance (ANOVA) followed by Tukey's post hoc test, with statistical significance defined as p < 0.05. Results are expressed as mean ± standard deviation (SD). In the figures, different lowercase letters (a, b, c, etc.) indicate statistically significant differences between groups, whereas identical letters denote no significant difference.

### 2.5 Analytical methods

Cell growth during shake flask fermentation was monitored by measuring the optical density at 600 nm (OD_600_). The quantification of vitamin B_6_, in the form of PN, was performed using high-performance liquid chromatography (HPLC). All measurements were carried out on a Thermo Fisher UltiMate® 3000 HPLC system equipped with a FLD-3400 fluorescence detector. Separation was achieved using a Cosmosil AR-II ODS column (250 mm × 4.6 mm, 5 μm particle size). The mobile phase A consisted of 33 mM phosphoric acid and 8 mM 1-octanesulfonic acid, with the pH adjusted to 2.4 using KOH. Mobile phase B consisted of 80 % (v/v) acetonitrile. The flow rate was maintained at 0.8 mL/min throughout the analysis. Prior to analysis, fermentation broth samples were centrifuged, and the supernatants were filtered through 0.22 μm membranes. Fluorescence detection was performed with an excitation wavelength of 293 nm and an emission wavelength of 395 nm. Quantification of PN was conducted using an external standard curve method. To ensure the accuracy and reproducibility of the measurements, the HPLC system was calibrated with standard solutions before each analysis. Samples were analyzed immediately after centrifugation or stored at −20 °C to prevent metabolite degradation. The developed HPLC method enables sensitive and reliable quantification of PN and provides strong analytical support for evaluating industrial vitamin B_6_ production [21].

## 3 Results and discussion

### 3.1 Chassis cell selection and medium selection

**Fig. 2:**
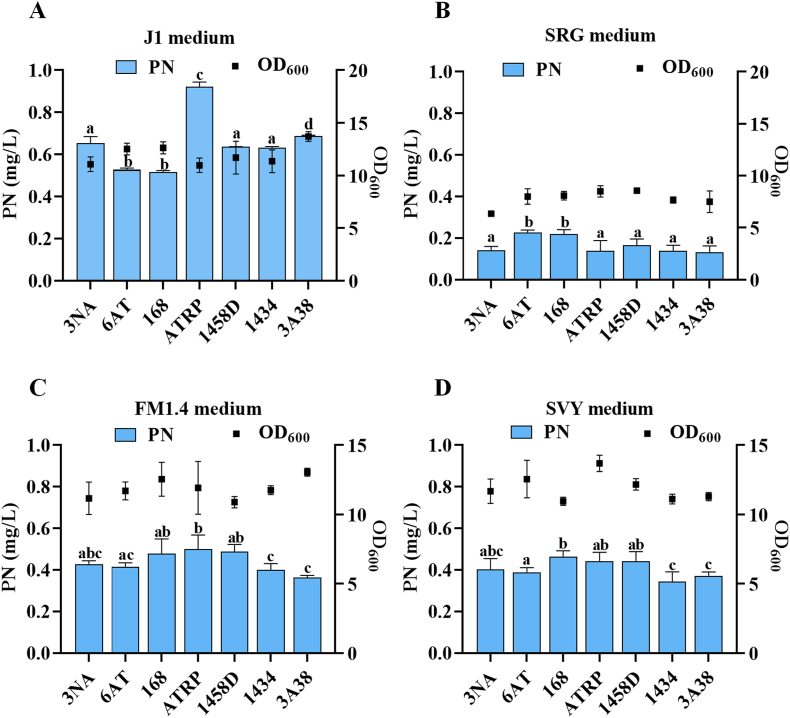
**Screening of the optimal chassis strain and medium for pyridoxine production**. Fermentation was carried out with *B. subtilis* 168, *B. subtilis* 3NA, *B. subtilis* 6AT, *B. subtilis* ATRP, *B. licheniformis* 1458D, *B. subtilis* 1434, and *B. subtilis* 3A38 strains, using four distinct media formulations: J1, SRG, FM1.4, and SVY. A. Results of shake flask fermentation of each strain using J1 medium. B. Results of shake flask fermentation of each strain using SRG medium. C. Results of shake flask fermentation of each strain using FM1.4 medium. D. Results of shake flask fermentation of each strain using SVY medium.

To identify a suitable chassis strain for PN production, seven representative *Bacillus* species were selected from the laboratory strain collection including *B. subtilis* 3NA, *B. subtilis* 6AT, *B. subtilis* 168, *B. subtilis* ATRP, *B. licheniformis* 1458D, *B. subtilis* 1434, and *B. subtilis* 3A38. These strains were cultivated in four distinct culture media, J1, SRG, FM1.4, and SVY, which were selected based on their composition and previous application in microbial production of PN [11,15,29]. To identify the peak PN yield, the strains were fermented in shake flasks using J1 medium for 72 h, with samples taken at 24, 48, and 72 h. The results revealed that the highest PN yield was achieved at 48 h (Fig. S1). Consequently, samples from the other three media were also collected at 48 h for further analysis. The PN production performance varied significantly depending on both the strain and the culture medium (Fig. 2). Among all tested combinations, *B. subtilis* ATRP cultivated in JI medium achieved the highest PN titer of 0.9 mg/L, indicating that this strain-medium pairing is optimal for further development (Fig. 2A). In contrast, SRG medium consistently resulted in the lowest PN yields for all strains, including ATRP (Fig. 2B). FM1.4 and SVY media supported moderate PN production, both showing relatively stable levels around 0.4 mg/L, with minimal variation across different strains (Fig. 2C and D). It should be noted that ATRP did not achieve the highest PN yield in every culture medium. However, it demonstrated the best overall performance, especially in J1 medium. The J1 medium provided the most favorable nutrient environment for ATRP, significantly enhancing PN production compared to other tested media due to its well-balanced composition of carbon sources, nitrogen sources, and essential cofactors.

As a result, *B. subtilis* ATRP grown in J1 medium was identified as the most promising combination for PN biosynthesis. However, the overall low PN titers observed among all tested laboratory strains highlight the limited native capacity of *Bacillus* species for PN production. Similarly, previous studies have reported that natural microbial strains generally exhibit low levels of PN synthesis [21,32,33]. This highlights the necessity of targeted metabolic engineering to improve PN production by strengthening the biosynthetic pathway involved in its synthesis.

### 3.2 Expression of the DXP-dependent pathway genes

**Fig. 3:**
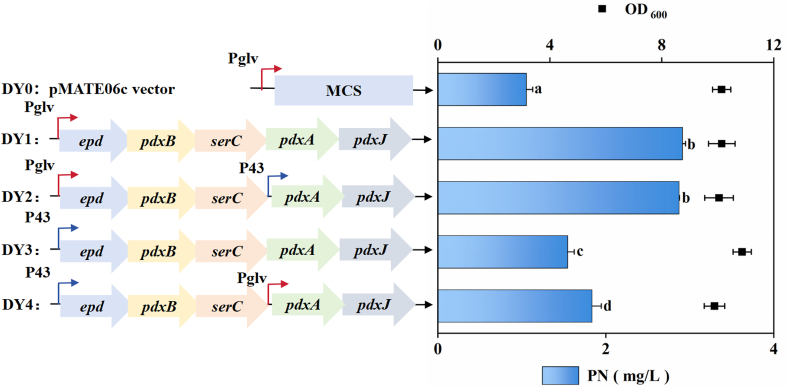
The PN titer of engineered strains by expressing the five genes of the DXP-dependent pathway in *B. subtilis* ATRP.

In our previous study, we successfully engineered an *E. coli* strain for efficient PN production via the DXP-dependent pathway by rationally optimizing key enzymes and enhancing precursor supply [29]. Among these, Epd was identified as a crucial enzyme for increasing the availability of E4P, a key precursor that drives carbon flux into the PN biosynthetic pathway. Expression of the *epd* gene from *Glaciecola nitratireducens* significantly enhanced PN production, yielding a 2.2-fold increase compared to the *epd* gene from *E. coli* due to improved NAD^+^ and E4P binding efficiencies. Furthermore, PdxA and PdxJ were confirmed to be rate-limiting enzymes due to their low catalytic efficiencies and high substrate K_m_ values [29]. Notably, their limited activity leads to the accumulation of the toxic intermediate 4HTP, which impairs cell growth. To address this, the expression of *pdxA* and *pdxJ* was increased, which not only relieved the metabolic bottleneck but also reduced 4HTP accumulation, thereby improving strain robustness and productivity. Based on these insights, the current study explores whether this optimized DXP-dependent pathway can function effectively in *B*. *subtilis*, an industrially favored host. By introducing the engineered pathway, we aim to assess the applicability of enhanced Epd-mediated precursor supply and PdxAJ-driven flux optimization. For this purpose, four distinct expression vectors were designed to modulate the expression of the gene cluster with P_glv_ [28] and P_43_ [34] promoters as illustrated in Fig. 3. We used GFP as a reporter gene and assessed the strength of the promoters by measuring the unit fluorescence intensity of the protein using a microplate spectrophotometer. The unit fluorescence intensity of the P_43_ promoter was 8,347, while that of the P_glv_ promoter reached up to 19,453. The vector employing the P_glv_ promoter to drive the entire cluster resulted in significantly higher PN production than the commonly used P_43_ promoter, consistent with the fact that P_glv_ exhibits stronger promoter activity than P_43_. Sampling during 24, 48 and 72 h of fermentation revealed that the highest titer was found at 48 h in Fig. S2. The highest titer achieved was 2.9 mg/L, representing a 3.2-fold improvement compared to the parental strain. However, further co-expression of PdxAJ under the P_43_ promoter did not increase PN titer. Overall, the observed low titer highlights a substantial metabolic incompatibility between the heterologous DXP-dependent pathway and the native metabolic network of *B. subtilis*.

These findings demonstrate that the expression of a DXP-dependent pathway for PN biosynthesis in *B*. *subtilis* leads to limited improvements in PN production. While functional expression of key enzymes such as PdxA and PdxJ enables PN synthesis via this non-native route, the overall titers remain low. According to literature reports, five genes encoding key enzymes of the non-native DXP-dependent biosynthesis pathway from *E. coli* and *Sinorhizobium meliloti* were assembled into two expression cassettes and integrated into the *B*. *subtilis* chromosome for functional expression [15,30]. Initial attempts without precursor supplementation resulted in PN titers around 14 mg/L. By co-feeding of intermediate metabolites such as 4HTP and DX, only limited improvement in PN production was observed under optimized conditions. This suggests that precursor availability alone cannot fully overcome the metabolic constraints associated with the heterologous pathway in *B. subtilis*. A key issue is that 4HTP serves not only as an intermediate in the pathway but also as an antimetabolite that inhibits the biosynthesis of essential amino acids such as threonine and isoleucine [15,35,36]. The accumulation of toxic intermediates, competition from native pathways, and stress-induced genetic instability collectively contribute to low productivity and strain fragility.

### 3.3 Screening for heterologous *pdxST* and gene RBS optimization

**Fig. 4:**
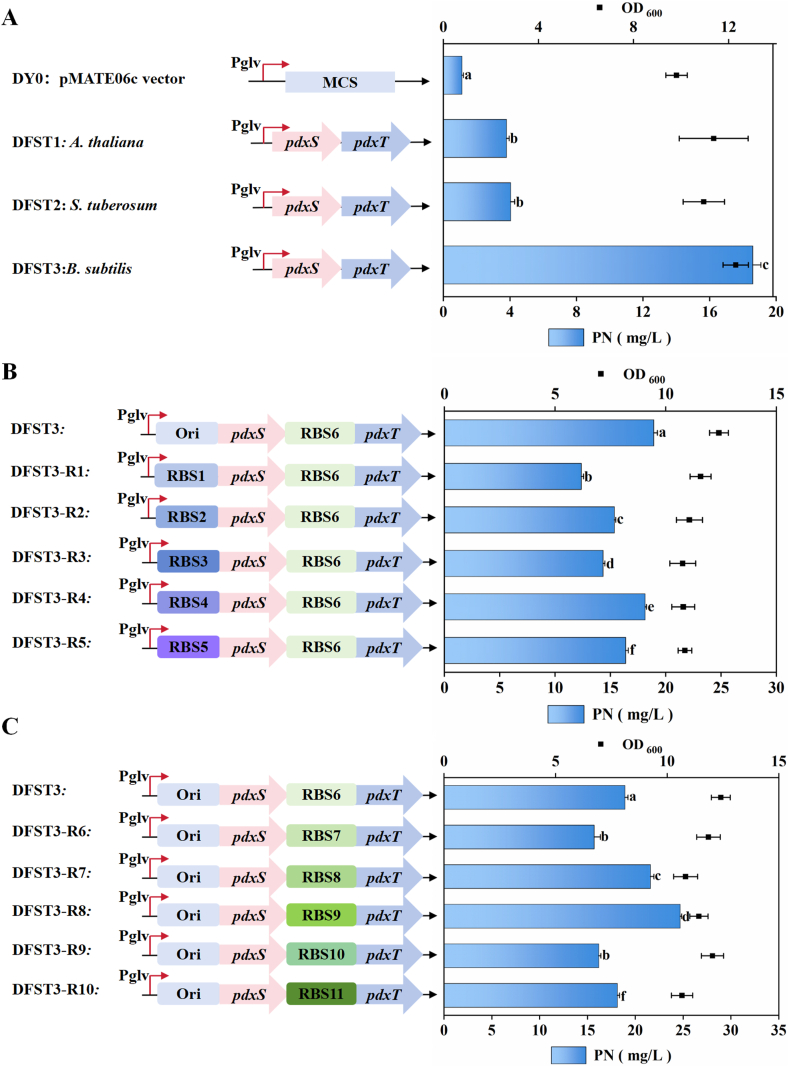
**The PN titer of engineered strains by expressing DXP-independent pathway genes *pdxST* in *B. subtilis* ATRP**. A. The PN titer of strains with overexpressed *pdxST* gene from different sources. B. The effect of regulating PdxS translation efficiency using different RBS sequences on PN production. C. The effect of regulating PdxT translation efficiency using different RBS sequences on PN production.

In light of the aforementioned results, we aim to further optimize PN biosynthesis by harnessing the DXP-independent pathway. Specifically, PdxS and PdxT, encoding the key enzymes of the PN salvage pathway, were introduced to catalyze the formation of PLP, the active form of vitamin B_6_, which is subsequently reduced to PN. To assess their effects on PN synthesis, *pdxST* genes from three different species, including *B*. *subtilis*, *Arabidopsis thaliana*, and *Solanum tuberosum*, were expressed in *B. subtilis* ATRP under the control of the P_glv_ promoter using the pMATE06c expression system. During shake flask fermentation, samples were taken at regular intervals to measure PN production. It was observed that PN yield continued to increase for the three strains even after 72 h. Consequently, the fermentation time was extended to 120 h. The highest PN yield was achieved at 96 h for the strains expressing the *pdxST* genes from three different sources, therefore, samples were taken at 96 h in all subsequent fermentations (Fig. S3). Comparative analysis showed that overexpression of the native *B. subtilis pdxST* genes led to the most significant increase in PN production, reaching 18.6 mg/L, whereas *pdxST* genes from *A. thaliana* and *S. tuberosum* resulted in limited improvements (Fig. 4A). These results suggest that the DXP-independent pathway is more compatible with the metabolic network of *B. subtilis* when driven by its native *pdxST* genes. To further improve PN production, the translation of PdxS and PdxT was optimized by engineering ribosome binding sites (RBSs). For PdxS, we tested five synthetic RBS variants, but none outperformed the native RBS provided by the pMATE06c plasmid. The highest titer of 18.6 mg/L was achieved using the native RBS, indicating it is already well-suited to the translational machinery of *B. subtilis* and likely ensures proper folding and expression balance within the PdxST complex (Fig. 4B). In contrast, RBS optimization of PdxT had a significant positive impact. Among six tested variants, the RBS with strength level 3 (RBS8) yielded the best results, increasing PN production to 24.7 mg/L, representing a 1.3-fold improvement over the baseline strain with unoptimized PdxT expression (Fig. 4C).

In previous studies, much of the effort toward PN biosynthesis has been centered on *E*. *coli*. Although the DXP-dependent PN pathway has been successfully reconstructed in *E. coli*, its implementation in *B*. *subtilis* has resulted in unsatisfactory PN titers, even after extensive metabolic engineering and medium optimization. One of the major limitations in *B. subtilis* arises from the intrinsic toxicity of the pathway intermediate 4HTP. By contrast, *B. subtilis* harbors a native *pdxST* operon, which provides a more compatible and efficient route for PN biosynthesis. Exploiting this endogenous pathway circumvents complications often associated with heterologous gene expression, such as protein misfolding and regulatory imbalance, and thus represents a host-adapted and metabolically robust strategy. To rigorously evaluate the relative performance of these two routes, we carried out a direct comparison of the DXP-dependent and *pdxST*-dependent pathways under identical genetic and cultivation conditions, employing the same promoter systems. The results demonstrated the superior compatibility of the native *pdxST* pathway in *B. subtilis*, which achieved higher PN titers without the accumulation of toxic intermediates.

### 3.4 Optimization of fermentation media

**Fig. 5:**
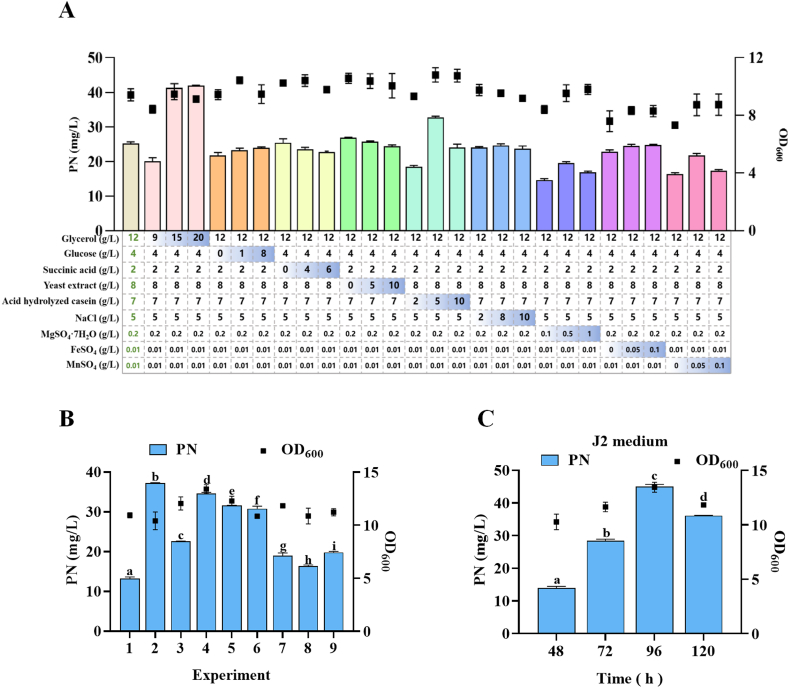
**Experimental optimization of PN production in DFST3-R8**. A. Single-factor of various culture medium components to assess their impact on PN yield. B. Orthogonal experimental design of four key culture medium factors (glycerol, acid hydrolyzed casein, MgSO4·7H_2_O, and MnSO_4_) resulting in nine distinct combinations, followed by fermentation to evaluate the corresponding PN production yields. C. Maximum pyridoxine yield after shaker fermentation of nine different combinations. D. Evaluation of the optimal combination from the orthogonal experimental design at fermentation time points of 48, 72, 96, and 120 h to identify the optimal fermentation time and maximum PN yield.

Despite the growing interest in microbial production of PN, there is a lack of systematic studies on medium optimization for *B*. *subtilis*-based PN biosynthesis. As the composition of the fermentation medium plays a pivotal role in microbial metabolism and product formation, a well-designed medium is essential for maximizing PN yield. To systematically optimize the fermentation conditions for PN production, the effects of various carbon source (glucose, glycerol, succinic acid), nitrogen source (yeast extract, acid hydrolyzed casein), metal ions (NaCl, MgSO_4_·7H_2_O, FeSO_4_, MnSO_4_) on PN titer were investigated using single-factor analysis (Fig. 5A). This preliminary screening revealed several key variables that significantly influenced PN biosynthesis. These variables were then subjected to an orthogonal array design to identify the optimal concentration combinations for improved PN production.

Based on the results of single-factor analysis, we identified the key components influencing PN production as glycerol (carbon source), acid hydrolyzed casein (nitrogen source), and the salts MgSO_4_·7H_2_O and MnSO_4_. Among these, the effects of carbon and nitrogen sources were particularly pronounced. Increasing the glycerol concentration to 15 g/L significantly enhanced PN production by 1.7-fold compared to the baseline, confirming that an ample carbon supply is critical for efficient biosynthesis (Fig. 5A). However, further increases beyond this concentration did not lead to additional improvements, indicating a saturation effect. Similarly, acid hydrolyzed casein showed an optimal concentration at 5 g/L, with both higher and lower levels resulting in decreased PN titers, suggesting that nitrogen source levels can influence intracellular metabolic flux distribution. The effects of various ions on PN production were also tested. Among the ions tested, MgSO_4_·7H_2_O and MnSO_4_ had a notable positive impact on PN production, while NaCl and FeSO_4_ showed negligible effects. This indicates that magnesium and manganese ions likely serve as essential cofactors for key enzymes in the PN biosynthetic pathway. After evaluating the effects of individual factors, the next step was to fix the concentrations of factors that had significant effects on PN titer.

To systematically determine the optimal concentrations and combinations of these factors, an orthogonal experimental design was employed. We selected glycerol, acid hydrolyzed casein, MgSO_4_·7H_2_O, and MnSO_4_ as variables for optimization and applied an L9(3^4^) orthogonal array, testing three levels of each component (Table S4). The nine combinations obtained were subjected to shake flask fermentation, and the PN yield is shown in Fig. 5B. Analysis of variance (ANOVA) of the experimental results shows that the factors polarities from smallest to largest are R(Acid hydrolyzed casein), R(MnSO_4_), R(MgSO_4_·7H_2_O), R(Glycerol) (Table S4). This indicates the primary to secondary importance of the factors in the following order: Glycerol, MgSO_4_·7H_2_O, MnSO_4_, and Acid hydrolyzed casein. Afterwards, the superior combination analysis revealed that Glycerol level 2, MgSO_4_·7H_2_O level 2, MnSO_4_ level 2, and Acid hydrolyzed casein level 2 were the superior combinations (Table S5). Subsequent optimal combination analysis identified the best combination as 15 g/L glycerol, 5 g/L acid hydrolyzed casein, 0.5 g/L MgSO_4_·7H_2_O, and 0.05 g/L MnSO_4_. Therefore, the optimal formulation was: glycerol 15 g/L, glucose 4 g/L, acid hydrolyzed casein 5 g/L, NaCl 5 g/L, MgSO_4_·7H_2_O 0.5 g/L, MnSO_4_ 0.05 g/L, FeSO_4_ 0.01 g/L and 100 mM Na_2_HPO_4_·12H_2_O. This combination formed the basis of the newly defined J2 medium. In the optimized J2 medium, the engineered *B. subtilis* strain produced up to 45.0 mg/L of PN, representing a 1.8-fold increase compared to the original J1 medium (Fig. 5C). This significant improvement in PN production highlights the effectiveness of medium optimization. The data confirmed that glycerol played the most pivotal role, likely due to its contribution to both energy metabolism and precursor supply for PN biosynthesis. While the variation in PN titer due to acid hydrolyzed casein was less pronounced, maintaining its concentration at an optimal level was still essential. Notably, interactions between magnesium and manganese ions contributed positively to PN production, likely by supporting cofactor-dependent enzymatic steps within the pathway. After optimizing the J2 medium, we further investigated the optimal temperature and pH for PN titer by the DFST3-R8 strain. Shake flask fermentation was conducted with adjustments to temperature and pH, and the highest PN yield was observed at 37 °C (Fig. S4A) and pH 6.5 (Fig. S4B).

### 3.5 Scale-up process culture in 5-L fermenter

**Fig. 6:**
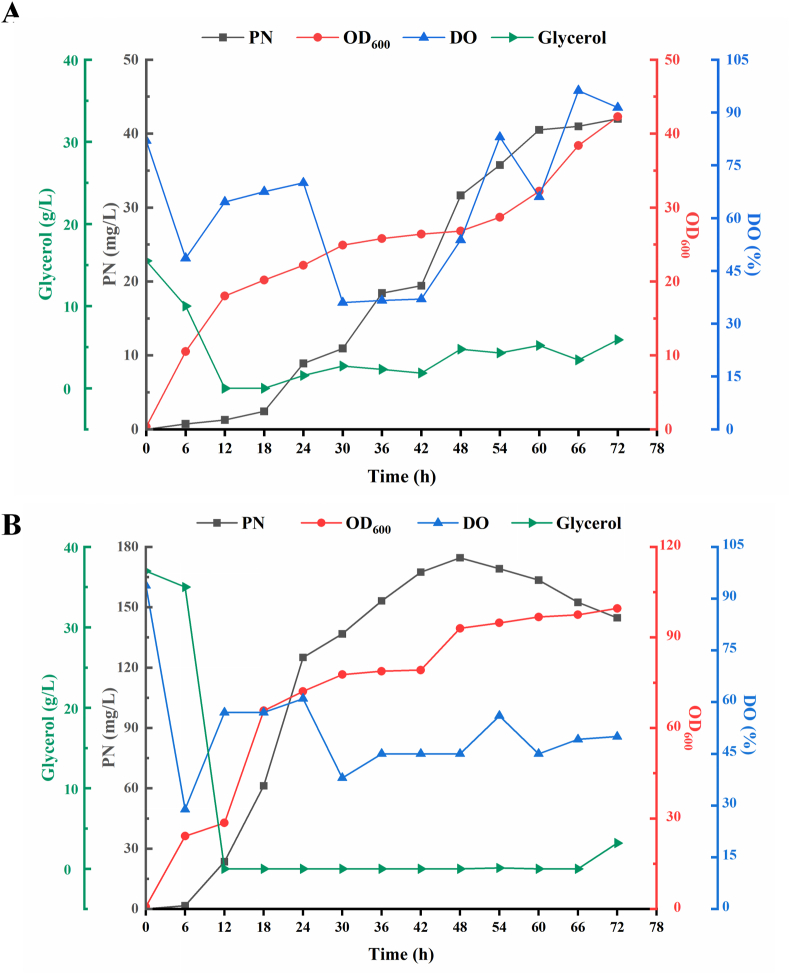
**Time course of fed-batch fermentation under different fermentation media**. A. The time course of PN concentration, OD_600_, and dissolved oxygen (DO%) during fed-batch fermentation using the J1 medium. B. The time course of PN concentration, OD_600_, and dissolved oxygen (DO%) during fed-batch fermentation using the J2 medium.

To validate the fermentation performance of the engineered *B*. *subtilis* strain, we conducted multiple fed-batch fermentations in J1 and J2 media in a 5-L bioreactor to compare their difference. The results showed that PN production was consistently higher in J2 medium than in J1; however, the maximum titer reached only 60.11 mg/L, indicating that the final yield was still relatively low (Fig. S5). After multiple fermentation attempts, we found that controlling the residual glycerol concentration in the fermentation broth was essential for improving PN production. Therefore, we finely controlled the glycerol feeding rate to ensure that the residual glycerol remained low. The J1 medium yielded 42.0 mg/L after optimizing the glycerol feeding rate, showing a 1.2-fold increase from its original titer of 34.56 mg/L (Fig. 6A). The residual glycerol concentration in the fermentation broth was difficult to control due to its slow consumption and the sluggish cell growth. In contrast, the PN titer in the bioreactor increased significantly in the J2 medium, reaching up to 174.6 mg/L, which was attributed to the improved control of glycerol concentration. (Fig. 6B). Notably, cell growth was also enhanced in the J2 medium, indicating improved metabolic activity and biomass accumulation. These results demonstrate that rational medium and process optimization significantly boost PN production in *B. subtilis*. The success of the J2 medium highlights its potential for industrial-scale application and provides a robust platform for the microbial production of PN.

## 4 Conclusions

In this study, we systematically engineered *B*. *subtilis* for efficient PN biosynthesis by optimizing both the metabolic pathway and gene expression, alongside tailoring the medium composition to support high-level production. Specifically, the enhancement of the DXP-independent pathway, combined with rational RBS optimization and strategic medium adaptation, resulted in a substantial increase in PN titer. In fed-batch fermentation using a 5-L bioreactor, the engineered strain achieved a remarkable PN titer of 174.6 mg/L, which, to the best of our knowledge, is the highest reported in *B. subtilis* to date. These findings not only provide a solid foundation for further optimization of *B. subtilis* strains but also pave the way for the development of scalable, high-yield processes for the commercial production of PN.

## CRediT authorship contribution statement

**Ai-Tong Jiang:** Writing – original draft, Methodology, Investigation, Conceptualization. **Guang-Qing Du:** Writing – review & editing, Writing – original draft, Methodology, Investigation, Funding acquisition, Conceptualization. **Xu-Yang Huang:** Writing – review & editing, Methodology. **Zheng-Zi Ji:** Writing – review & editing. **Si-Riguleng Qian:** Writing – review & editing, Resources, Funding acquisition, Conceptualization. **Lin-Xia Liu:** Writing – review & editing, Resources, Funding acquisition, Conceptualization. **Da-Wei Zhang:** Writing – review & editing, Supervision, Resources, Funding acquisition, Conceptualization.

## Funding

This work was supported by the National Key R&D Program of China (2022YFC2106100), the 10.13039/501100001809National Natural Science Foundation of China (22178372, 22208368, 32200049), the National Science Fund for Distinguished Young Scholars (22325807), the International Partnership Program of the Chinese Academy of Sciences (306GJHZ2023019GC), and the Tianjin Synthetic Biotechnology Innovation Capacity Improvement Project (TSBICIP-CXRC-070).

## **Declaration of competing interest**

The authors declare that they have no known competing financial interests or personal relationships that could have appeared to influence the work reported in this paper.
