# Supplementary material for: Enhanced microbial production of pyridoxine (Vitamin B6) in Bacillus subtilis via pathway and process optimization
Source: Synth Syst Biotechnol. 2025 Sep 16;11:216–25. doi: 10.1016/j.synbio.2025.09.014 (PMC12517072; doi:10.1016/j.synbio.2025.09.014)
Supplement: Multimedia component 1 [file mmc1.docx]

**Supplementary material:**

**Enhanced Microbial Production of Pyridoxine (Vitamin B_6_) in *Bacillus subtilis* via Pathway and Process Optimization**

Ai-Tong Jiang^a,b,c,d,1^, Guang-Qing Du^b,c,d,1^, Xu-Yang Huang^a,b,c,d^, Zheng-Zi Ji^b,c,d^, Lin-Xia Liu ^b,c,d,e*^, Si-Riguleng Qian^a,**^, Da-Wei Zhang^b,c,d,e*^

^a^ School of Biological Engineering, Dalian Polytechnic University, Dalian, China.

^b^ Tianjin Institute of Industrial Biotechnology, Chinese Academy of Sciences, Tianjin, China.

^c^ National Technology Innovation Center of Synthetic Biology, Tianjin, China.

^d^ State Key Laboratory of Engineering Biology for Low-Carbon Manufacturing, Tianjin

Institute of Industrial Biotechnology, Chinese Academy of Sciences, Tianjin, China.

^e^ University of Chinese Academy of Sciences, Beijing, China.

**Correspondence:**

** Siriguleng Qian, E-mail: srgl2000@163.com.

* Lin-Xia Liu, E-mail: [liulx@tib.cas.cn](mailto:liulx@tib.cas.cn).

* Dawei Zhang, E-mail: zhang_dw@tib.cas.cn.

^1^ These authors contributed equally to this work

**Table S1.** Strains and plasmids used in this work

| **Strain** | **Description** | **Source** |
| --- | --- | --- |
| *E. coli* DH5α | Host for the construction of plasmids | Invitrogen |
| *B. subtilis* 168 | Wild type | This lab |
| *B. subtilis* 3A38 | Wild type | This lab |
| *B. subtilis* 3NA | Wild type | This lab |
| *B. subtilis* 1434 | Wild type | This lab |
| *B. subtilis* ATRP | Wild type | This lab |
| *B. subtilis* 6AT | Wild type | This lab |
| *B. licheniformis* 1458D | Wild type | This lab |
| DY0 | *B. subtilis* ATRP carrying pMATE06c vector | This work |
| DY1 | Simultaneous expression of *epd, pdxB, serC, pdxA,* and *pdxJ* genes with pMATE06c in *Bacillus subtilis* ATRP | This work |
| DY2 | Simultaneous expression of *epd, pdxB, serC,* pdxA, and *pdxJ* genes with pMATE06c, where *pdxA*, *pdxJ* are regulated using the P_43_ promoter | This work |
| DY3 | Simultaneous expression of *epd, pdxB, serC, pdxA,* and *pdxJ* genes with pMATE06c in *Bacillus subtilis* ATRP, where the original promoter replaced with P_43_ promoter | This work |
| DY4 | Simultaneous expression of *epd, pdxB* and *serC* genes with pMATE06c, where *epd, pdxB* and *serC* are regulated using the P_43_ promoter | This work |
| DFST1 | Simultaneous expression of *pdxS* and *pdxT* genes with pMATE06c in *Bacillus subtilis* ATRP, including the *pdxST* gene was derived from *Arabidopsis thaliana* | This work |
| DFST2 | Simultaneous expression of *pdxS* and *pdxT* genes with pMATE06c in *Bacillus subtilis* ATRP, including the *pdxST* gene was derived from *Solanum tuberosum* | This work |
| DFST3 | Simultaneous expression of *pdxS* and *pdxT* genes with pMATE06c in *Bacillus subtilis* ATRP, including the *pdxST* gene was derived from *Bacillus subtilis* | This work |
| DFST3-R1 | RBS of *pdxS* in pMATE06c plasmid was replaced with RBS 5851.15 | This work |
| DFST3-R2 | RBS of *pdxS* in pMATE06c plasmid was replaced with RBS 8390.33 | This work |
| DFST3-R3 | RBS of *pdxS* in pMATE06c plasmid was replaced with RBS 10112.81 | This work |
| DFST3-R4 | RBS of *pdxS* in pMATE06c plasmid was replaced with RBS 26256.10 | This work |
| DFST3-R5 | RBS of *pdxS* in pMATE06c plasmid was replaced with RBS 46915.26 | This work |
| DFST3-R6 | RBS of *pdxT* in pMATE06c plasmid was replaced with RBS 5454.20 | This work |
| DFST3-R7 | RBS of *pdxT* in pMATE06c plasmid was replaced with RBS 8577.02 | This work |
| DFST3-R8 | RBS of *pdxT* in pMATE06c plasmid was replaced with RBS 9752.6 | This work |
| DFST3-R9 | RBS of *pdxT* in pMATE06c plasmid was replaced with RBS 24477.06 | This work |
| DFST3-R10 | RBS of *pdxT* in pMATE06c plasmid was replaced with RBS 44377.87 | This work |
| **Plasmids** | **Description** | **Source** |
| pP43NMK | AMP, Kana, *E. coli*-*B. subtilis* shuttle vector | [3] |
| pMATE06c-Pglv-epd-pdxB-serC | pMATE06c derivative expressing *epd,* *pdxB,* *serC* from *E. coli* | This work |
| pP43NMK-pdxA-pdxJ | pP43NMK derivative expressing *pdxA,* *pdxJ* from *E. coli* | This work |
| pP43NMK-epd-pdxB-serC | pP43NMK derivative expressing *epd,* *pdxB,* *serC* from *E. coli* | This work |
| pMATE06c-pdxA-pdxJ | pMATE06c derivative expressing *pdxA,* *pdxJ* from *E. coli* | This work |
| pMATE06c-Pglv-epd-pdxB-serC-P43-pdxA-pdxJ | pMATE06c derivative expressing *epd,* *pdxB,* *serC, pdxA and pdxJ,* where pdxA, pdxJ are regulated using the P_43_ promoter | This work |
| pMATE06c-Pglv-epd-pdxB-serC-pdxA-pdxJ | pMATE06c derivative expressing *epd,* *pdxB,* *serC, pdxA and pdxJ* | This work |
| pMATE06c-PdxST-Bs | pMATE06c derivative expressing *pdxS,* *pdxT* from *E. coli*, including the *pdxST* gene was derived from *Bacillus subtilis* | This work |
| pMATE06c-PdxST-AT | pMATE06c derivative expressing *pdxS,* *pdxT* from *E. coli,* including the *pdxST* gene was derived from *Arabidopsis thaliana* | This work |
| pMATE06c-PdxST-ST | pMATE06c derivative expressing *pdxS,* *pdxT* from *E. coli,* including the *pdxST* gene was derived from *Solanum tuberosum* | This work |
| pMATE06c-PdxST-Bs-R1 | pMATE06c-PdxST-Bs derivate, RBS of *pdxS* was replaced with RBS 5851.15 | This work |
| pMATE06c-PdxST-Bs-R2 | pMATE06c-PdxST-Bs derivate, RBS of *pdxS* was replaced with RBS 8390.33 | This work |
| pMATE06c-PdxST-Bs-R3 | pMATE06c-PdxST-Bs derivate, RBS of *pdxS* was replaced with RBS 10112.81 | This work |
| pMATE06c-PdxST-Bs-R4 | pMATE06c-PdxST-Bs derivate, RBS of *pdxS* was replaced with RBS 26256.10 | This work |
| pMATE06c-PdxST-Bs-R5 | pMATE06c-PdxST-Bs derivate, RBS of *pdxS* was replaced with RBS 46915.26 | This work |
| pMATE06c-PdxST-Bs-R6 | pMATE06c-PdxST-Bs derivate, RBS of *pdxT* was replaced with RBS 5454.20 | This work |
| pMATE06c-PdxST-Bs-R7 | pMATE06c-PdxST-Bs derivate, RBS of *pdxT* was replaced with RBS 8577.02 | This work |
| pMATE06c-PdxST-Bs-R8 | pMATE06c-PdxST-Bs derivate, RBS of *pdxT* was replaced with RBS 9752.6 | This work |
| pMATE06c-PdxST-Bs-R9 | pMATE06c-PdxST-Bs derivate, RBS of *pdxT* was replaced with RBS 24477.06 | This work |
| pMATE06c-PdxST-Bs-R10 | pMATE06c-PdxST-Bs derivate, RBS of *pdxT* was replaced with RBS 44377.87 | This work |

**Table S2.** The primers used in this work

| **Name** | **Sequence（5’to 3’）** |
| --- | --- |
| epd-pdxB-F | GAAGGAGGTCGTCAAATGGCACACCGCATTGCGATT |
| epd-pdxB-R | GTTTGTTTAATATCGTCTAACGAGCCGGGTGGTGAAC |
| serC-F | GCTCGTTAGACGATATTAAACAAACGTAACGAGTTATTTTATGG |
| serC-R | GTCGGAACGAGACTTCTTAACCGTGACGGCGTTCGA |
| pdxAJ-F | TAGCGGTACCCAGACATTTCTCTTAAGGAGGTTTTTTATG |
| pdxAJ-R | CCAAGCTTCTGCAGTTAGCCACGCGCTTCCAGCA |
| pdxAJ-2F | CGTCACGGTTAACAGACATTTCTCTTAAGGAGGTTTTTTATGG |
| PdxAJ-2R | CGGAACGAGACTTCTTAGCCACGCGCTTCCAGCA |
| P-epd-pdxB-serC-F | GCCGTCACGGTTAAGAAGTCTCGTTCCGACAGTTGG |
| P-epd-pdxB-serC-R | CGGTGTGCCATTTGACGACCTCCTTCTATTTAAAAACAATTCC |
| P-pdxAJ-F | GAAGCGCGTGGCTAACTGCAGAAGCTTGGCGTAATC |
| P-pdxAJ-R | TTAAGAGAAATGTCTGGGTACCGCTATCACTTTATATTTTACATAATCG |
| P-P43-pdxAJ-2R | CATACCACCTATCATTAACCGTGACGGCGTTCGA |
| P-P43-pdxAJ-2F | CCGTCACGGTTAATGATAGGTGGTATGTTTTCGCTTGAAC |
| P43-epd-pdxB-serC-F | AGTGATAGCGGTACCTTTTTAAATAGAAGGAGGTCGTCAAATGG |
| P43-epd-pdxB-serC-R | ACCTCCTTAAGAGAAATGTCTGGACGGCGTTCGAA |
| pdxAJ-3F | TTCGAACGCCGTCCAGACATTTCTCTTAAGGAGGTTTTTTATG |
| pdxAJ-3R | TATTCTCATTCCAACTGTCGGAACGAGACTTCTAGCCACGCGCTTCCAGC |
| Pglv-pdxAJ-F | GAAGGAGGTCGTCAACAGACATTTCTCTTAAGGAGGTTTTTTATG |
| Pglv-pdxAJ-R | GTCGGAACGAGACTTCTTAGCCACGCGCTTCCAGCA |
| BS-pdxS-F | TTTATCAAGGAGGTCGTCATATGGCTCAAACAGGTACTGAACG |
| BS-pdxS-R | AACTTACCAGCCGCGTTCTTGC |
| BS-pdxT-F | AAGAACGCGGCTGGTAAGTTGGGCCGCAAAGGAGG |
| BS-pdxT-R | GGAACGAGACTTCTTAATTATTATACAAGTGCCTTTTGCTTATATTCC |
| AT-pdxST-F | ATCAAGGAGGTCGTCATATGGCAGGGACAGGAG |
| AT-pdxST-R | ACTGTCGGAACGAGACTTCTTAATTATTACTCGGAACGGCTCG |
| ST-pdxST-F | CAAGGAGGTCGTCATATGGCTGGATCAGGGG |
| ST-pdxST-R | CTACAAACTGCATAACTCATTTATTGGTAGATCGGAAAATCAATAATG |
| Pmate06c-cp-F | TATGGTTTTGGTCGGCACTGCC |
| Pmate06c-cp-R | TGCATCCGCTTACAGACAAGC |
| DFST3-R1-F | TGGCTAAGGATCACTAAAAAGAAAAAATTAATGGCTCAAACAGGTACTGAAC |
| DFST3-R1-R | ATTAATTTTTTCTTTTTAGTGATCCTTAGCCAATTCCATTTATACCATGAGATAGC |
| DFST3-R2-F | TTGTTGGCATAGACAGGAATCTATAGCATGGCTCAAACAGGTACTGAAC |
| DFST3-R2-R | CCATGCTATAGATTCCTGTCTATGCCAACAATTCCATTTATACCATGAGATAGCTC |
| DFST3-R3-F | GAATTGAATTTTAAAAGGTATTATCAACAAATGGCTCAAACAGGTACTGAACG |
| DFST3-R3-R | CATTTGTTGATAATACCTTTTAAAATTCAATTCCATTTATACCATGAGATAGCT |
| DFST3-R4-F | TTGTAACTAATAAATTGAGGTCTTTATATGGCTCAAACAGGTACTGAAC |
| DFST3-R4-R | CATATAAAGACCTCAATTTATTAGTTACAATTCCATTTATACCATGAGATAGCTC |
| DFST3-R5-F | GTCTATTAAGATAAGTAGGATTTTTAAATGGCTCAAACAGGTACTGAAC |
| DFST3-R5-R | ATTTAAAAATCCTACTTATCTTAATAGACAATTCCATTTATACCATGAGATAGCTC |
| DFST3-R6-F | TAATTAAGACAGAAACGAAATACTTTTATGTTAACAATAGGTGTACTAGGACTTC |
| DFST3-R6-R | AACATAAAAGTATTTCGTTTCTGTCTTAATTACCAGCCGCGTTCTTGCA |
| DFST3-R7-F | GTAATGTATAATTAACGAGAATACAAAATGTTAACAATAGGTGTACTAGGACTTC |
| DFST3-R7-R | TAACATTTTGTATTCTCGTTAATTATACATTACCAGCCGCGTTCTTGCA |
| DFST3-R8-F | GTAAGTTGGGCCGCAAAGGAGGTAAAAAAATGTTAACAATAGGTG |
| DFST3-R8-R | GTTAACATTTTTTTACCTCCTTTGCGGCCCAACTTACCAGCCGCGTTC |
| DFST3-R9-F | GGTAACGGTTAGAAAGAGAGAATTTAAGATGTTAACAATAGGTGTACTAGGACTTC |
| DFST3-R9-R | TTAACATCTTAAATTCTCTCTTTCTAACCGTTACCAGCCGCGTTCTTGCA |
| DFST3-R10-F | GGTAAATAGAAATAAAAGGATTATAAATTATGTTAACAATAGGTGTACTAGGACTTC |
| DFST3-R10-R | TAACATAATTTATAATCCTTTTATTTCTATTTACCAGCCGCGTTCTTGCA |

**Table S3.** RBS Sequences used to express *pdxS* and *pdxT* genes

| **Name** | **Predicted translation initiation rate** | **Sequence** |
| --- | --- | --- |
| RBS1 | 5851.15 | CACTAAAGAAAGTAGAAAATATAATA |
| RBS2 | 8390.33 | GCTAAGGATCACTAAAAAGAAAAAATTA |
| RBS3 | 10112.81 | TTGGCATAGACAGGAATCTATAGC |
| RBS4 | 26256.1 | AATTTTAAAAGGTATTATCAACAA |
| RBS5 | 46914.26 | TAACTAATAAATTGAGGTCTTTAT |
| RBS6 | 10429.89 | TCTATTAAGATAAGTAGGATTTTTAA |
| RBS7 | 5454.20 | TTAAGACAGAAACGAAATACTTTT |
| RBS8 | 8577.02 | TGTATAATTAACGAGAATACAAA |
| RBS9 | 9752.6 | GTTGGGCCGCAAAGGAGGTAAAAAA |
| RBS10 | 44377.87 | CGGTTAGAAAGAGAGAATTTAAG |
| RBS11 | 88425.90 | ATAGAAATAAAAGGATTATAAATT |

**Table S4.** Factors in orthogonal experiments and specific levels for each factor

| **Factors** | **Glycerol (g/L)** | **Acid hydrolyzed casein (g/L)** | **MgSO_4_·7H_2_O (g/L)** | **MnSO_4_ (g/L)** | **PN Production** |
| --- | --- | --- | --- | --- | --- |
| Experiment 1 | 12 | 2 | 0.2 | 0.01 | 13.2020 |
| Experiment 2 | 12 | 5 | 0.5 | 0.05 | 37.2162 |
| Experiment 3 | 12 | 7 | 1 | 0.1 | 22.6346 |
| Experiment 4 | 15 | 2 | 0.5 | 0.1 | 34.6431 |
| Experiment 5 | 15 | 5 | 1 | 0.01 | 31.6340 |
| Experiment 6 | 15 | 7 | 0.2 | 0.05 | 30.8242 |
| Experiment 7 | 20 | 2 | 1 | 0.05 | 19.0167 |
| Experiment 8 | 20 | 5 | 0.2 | 0.1 | 16.3628 |
| Experiment 9 | 20 | 7 | 0.5 | 0 | 19.8230 |
| Mean 1 | 24.351 | 22.287 | 20.126 | 21.553 |  |
| Mean 2 | 32.364 | 28.404 | 30.561 | 29.016 |  |
| Mean 3 | 18.401 | 24.424 | 24.428 | 24.547 |  |
| Range | 13.963 | 6.117 | 10.435 | 7.463 |  |

**Table S5.** Optimal combination analysis

| **considerations** | **A** | **B** | **C** | **D** |
| --- | --- | --- | --- | --- |
| K1 | 24.351 | 22.287 | 20.126 | 21.553 |
| K2 | 32.364 | 28.404 | 30.561 | 29.016 |
| K3 | 18.401 | 24.424 | 24.428 | 24.547 |
| k1 | 8.117 | 7.429 | 6.709 | 7.184 |
| k2 | 10.788 | 9.468 | 10.187 | 9.672 |
| k3 | 6.134 | 8.141 | 8.143 | 8.182 |


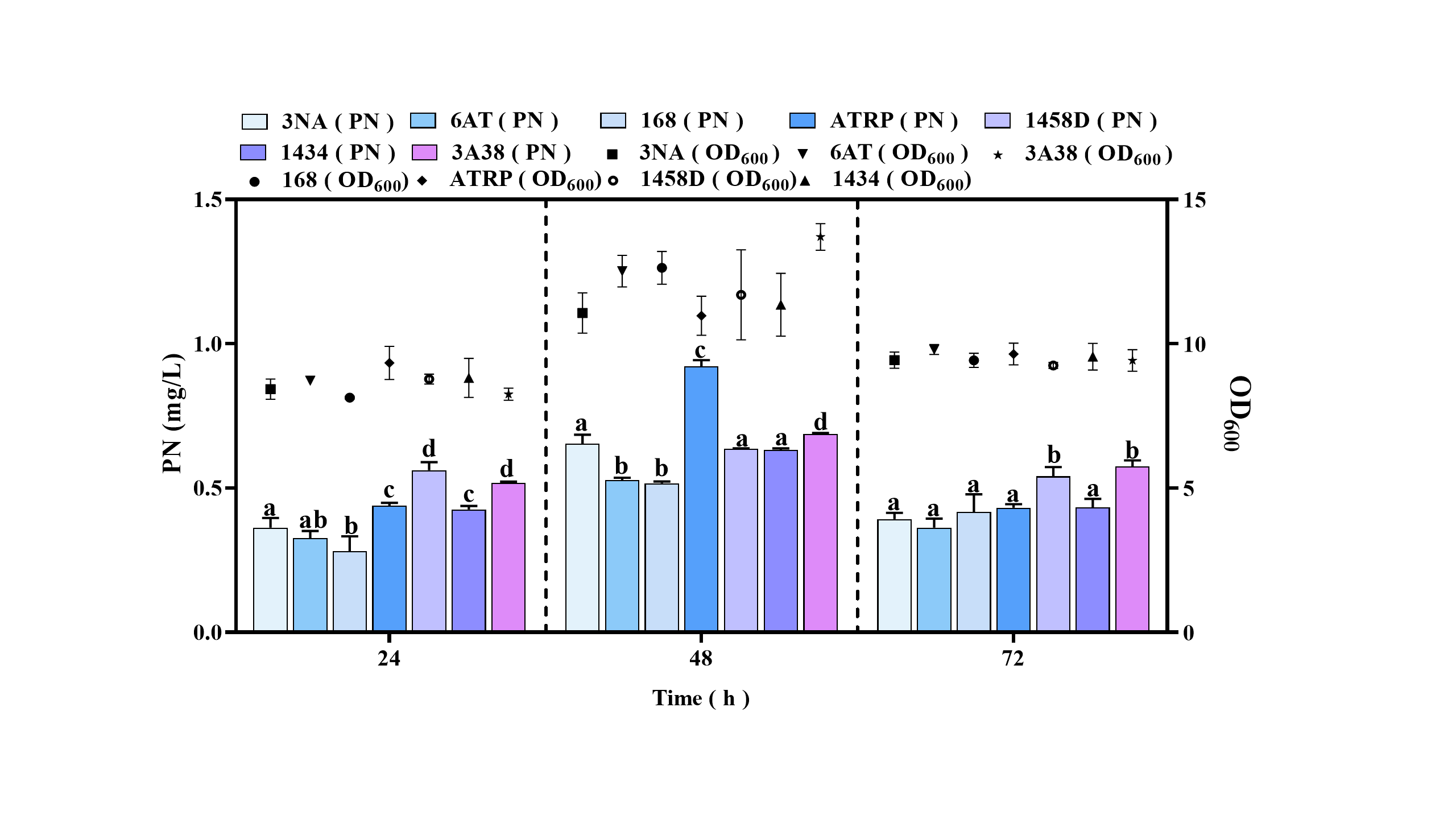


**Fig S1:** **PN production profiles of wild-type Bacillus strains in J1 medium over 24, 48, and 72 h of shake-flask fermentation.** PN production was measured for B. subtilis 168, B. subtilis 3NA, B. subtilis 6AT, B. subtilis ATRP, B. licheniformis 1458D, B. subtilis 1434, and B. subtilis 3A38. The data show strain-specific variations in PN production at different time points (24, 48, and 72 h), with distinct yield trends observed across strains, indicating differences in metabolic capacity and biosynthetic efficiency.

**
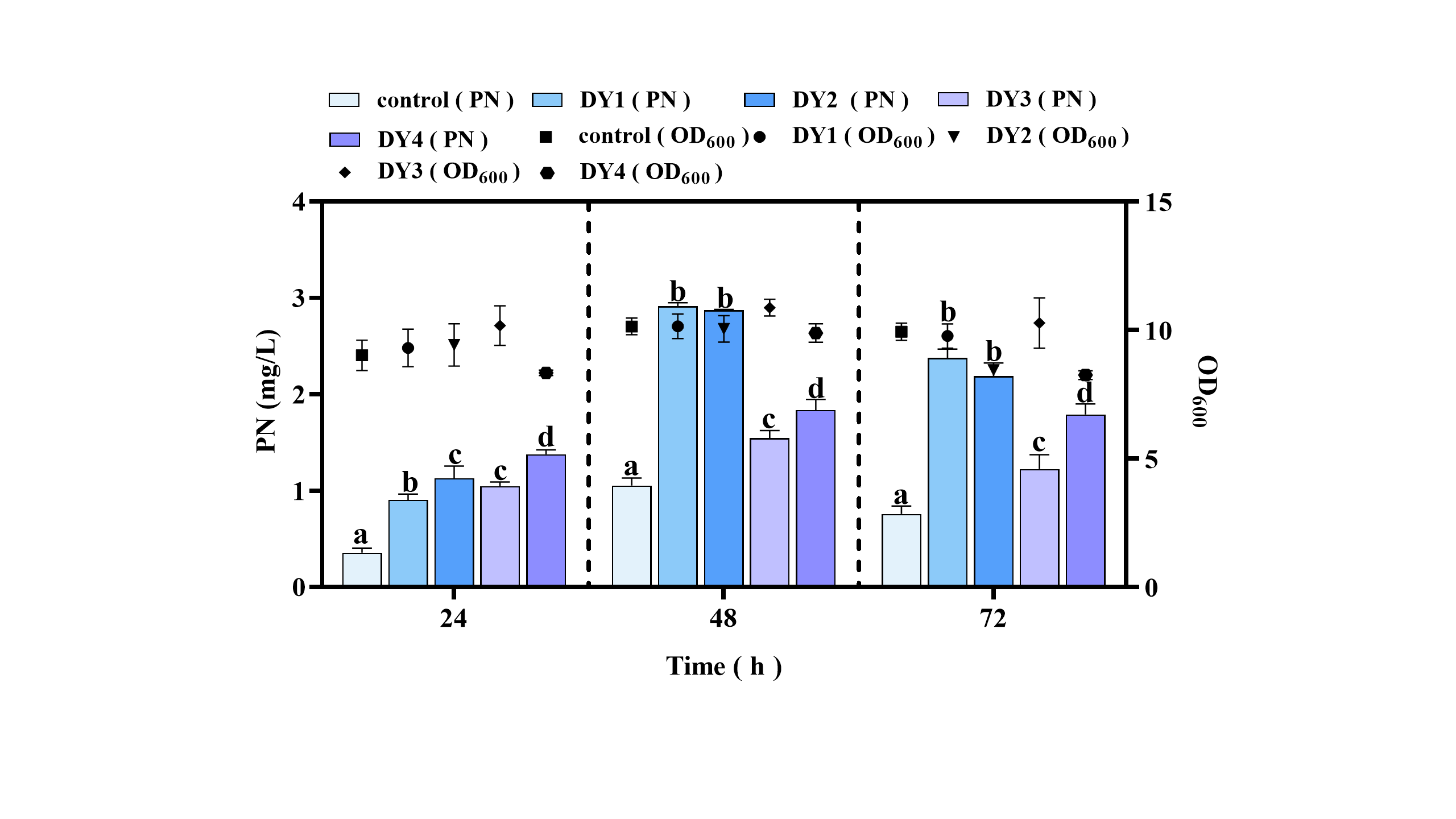
**

**Fig S2:** PN production by genetically engineered B. subtilis ATRP expressing five DXP-dependent pathway genes, with four derived strains: DY1, DY2, DY3, and DY4, measured at 24, 48, and 72 h.


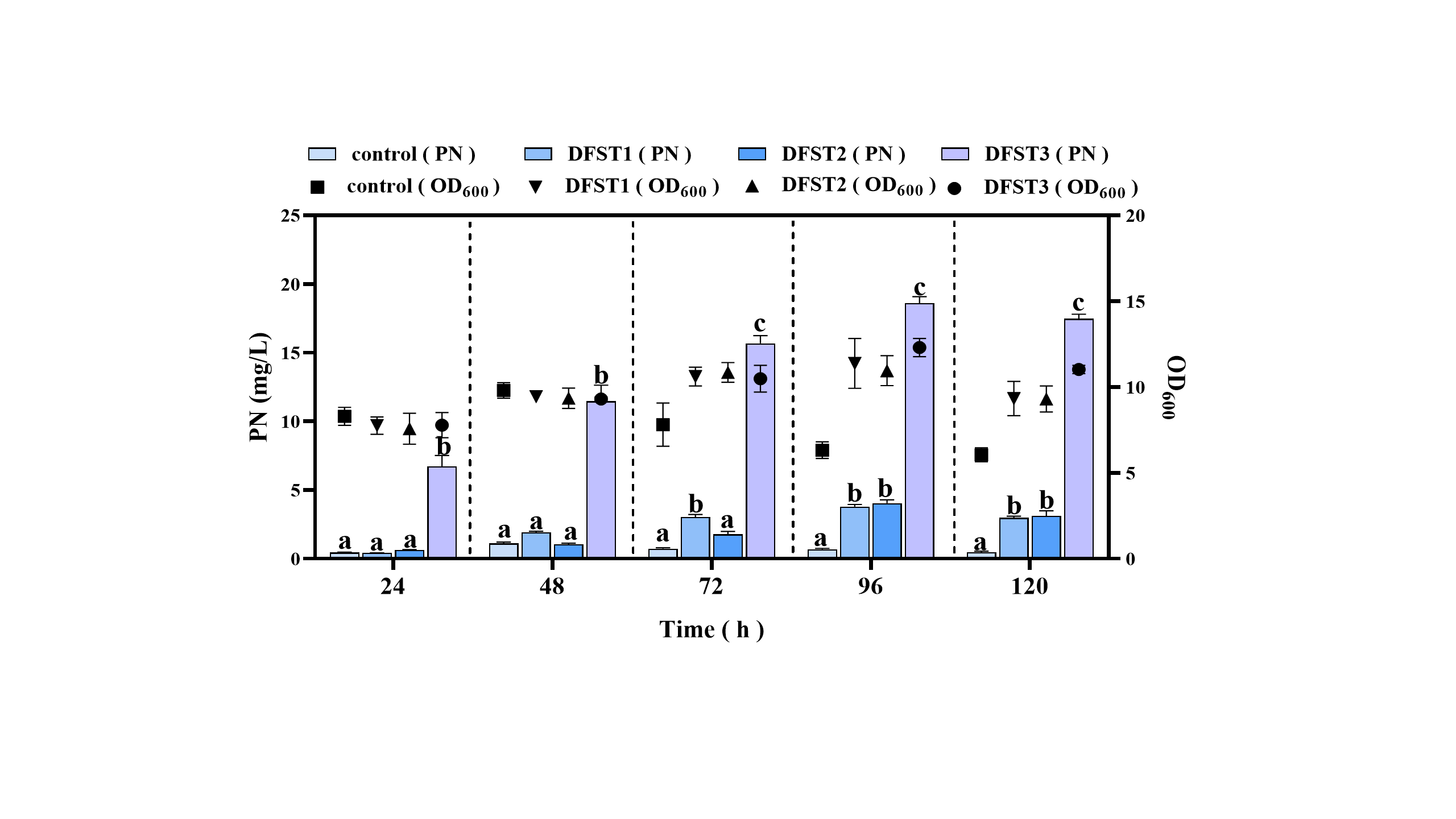


**Fig S3:** PN yield in shake flask fermentation of B. subtilis ATRP strains expressing *pdxST* genes from different sources at various time points (24, 48, 72, 96, and 120 hours). The data show the temporal variation in PN production as influenced by the expression of the DXP-independent pathway genes. PN yield was measured at each time point to determine the optimal production period.


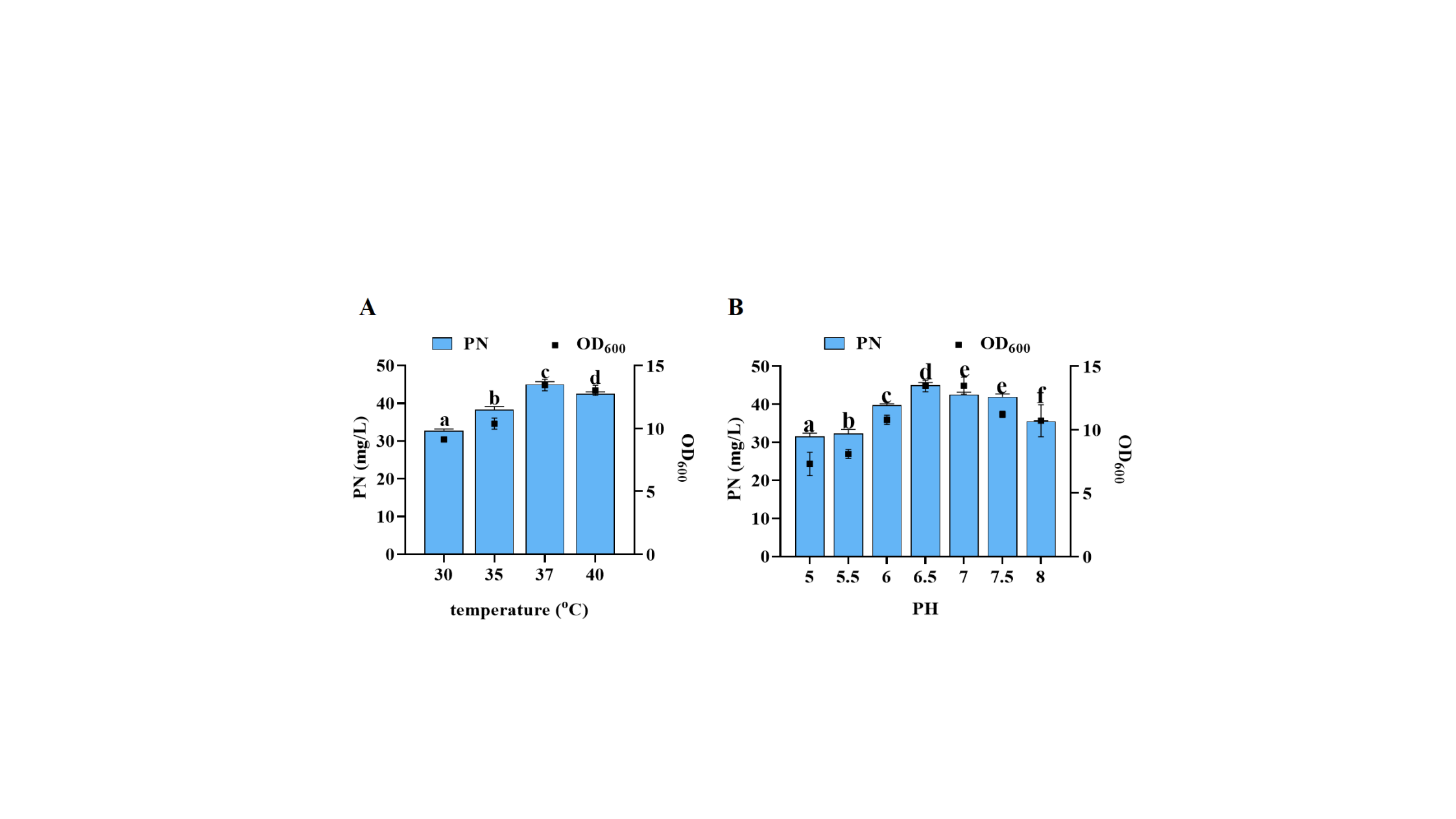


**Fig S4: Optimization of fermentation temperature and pH for PN production in B. subtilis DFST3-R8.** A. PN yield was measured at different temperatures, highlighting the optimal temperature for maximum product synthesis. B. PN production was evaluated at different pH levels (5.0, 5.5, 6.0, 6.5, 7.0, 7.5, and 8.0) of shake-flask fermentation. The results demonstrate the effect of pH on PN production, with an optimal pH identified that maximizes both microbial growth and enzymatic activity involved in PN biosynthesis.


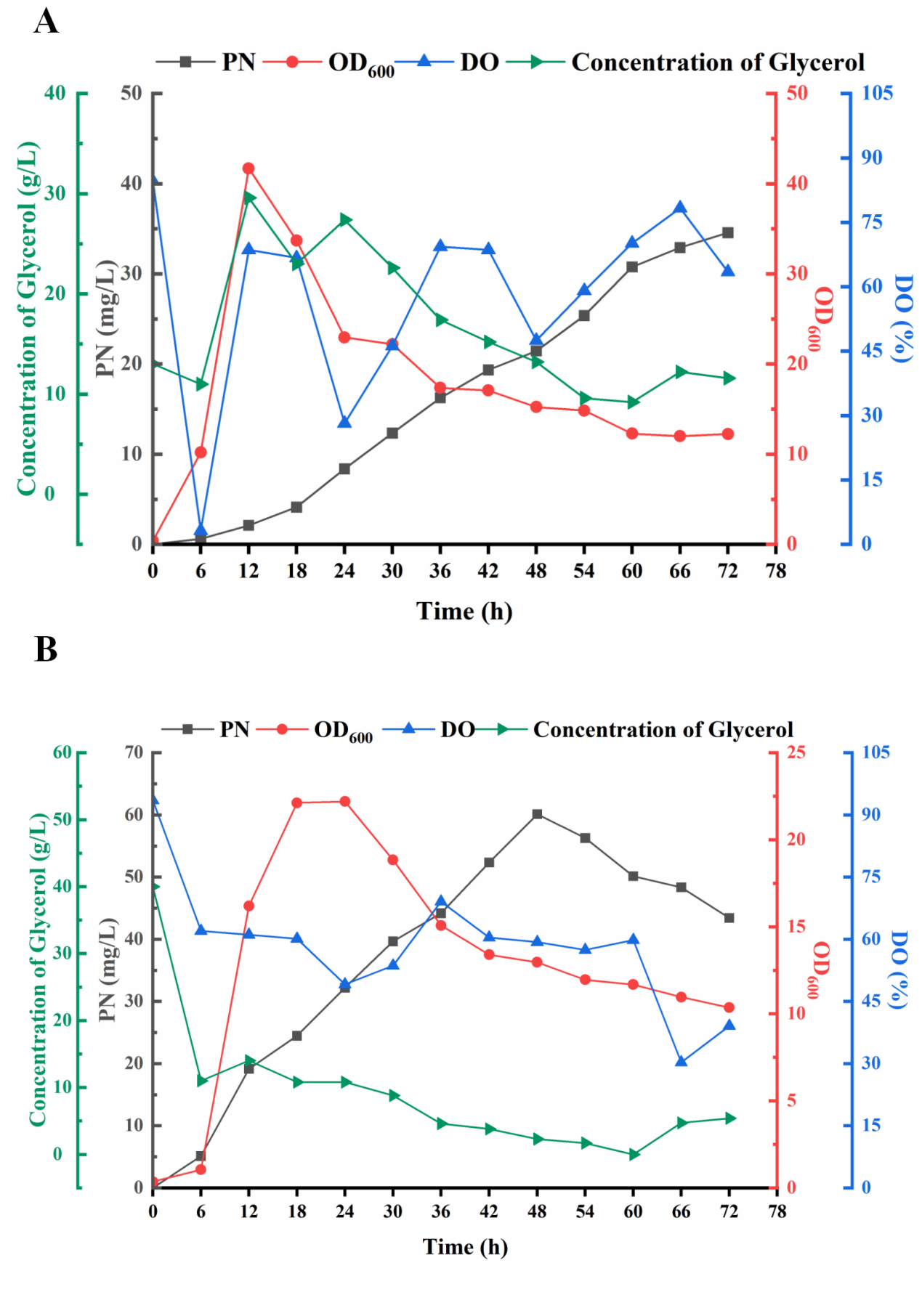


**Fig S5: Time course of fed-batch fermentation under different fermentation media.** A. The time course of PN concentration, OD_600_, and dissolved oxygen (DO%) during fed-batch fermentation using the J1 medium. B. The time course of PN concentration, OD_600_, and dissolved oxygen (DO%) during fed-batch fermentation using the J2 medium.
